# Identification of distinct subpopulations of *Gli1‐*lineage cells in the mouse mandible

**DOI:** 10.1111/joa.13858

**Published:** 2023-03-10

**Authors:** Nian Zhang, William B. Barrell, Karen J. Liu

**Affiliations:** ^1^ Centre for Craniofacial and Regenerative Biology, Faculty of Dentistry, Oral and Craniofacial Sciences King's College London London UK; ^2^ State Key Laboratory of Oral Disease, Department of Oral and Maxillofacial Surgery, National Clinical Research Center for Oral Diseases West China Hospital of Stomatogy, Sichuan University Chengdu China

**Keywords:** *Gli1*, mandibular development, mouse, neural crest, skeletal stem cells

## Abstract

The Hedgehog pathway gene *Gli1* has been proposed to mark a subpopulation of skeletal stem cells (SSCs) in craniofacial bone. Skeletal stem cells (SSCs) are multi‐potent cells crucial for the development and homeostasis of bone. Recent studies on long bones have suggested that skeletal stem cells in endochondral or intramembranous ossification sites have different differentiation capacities. However, this has not been well‐defined in neural crest derived bones. Generally, the long bones are derived from mesoderm and follow an endochondral ossification model, while most of the cranial bones are neural crest (NC) in origin and follow an intramembranous ossification model. The mandible is unique: It is derived from the neural crest lineage but makes use of both modes of ossification. Early in fetal development, the mandibular body is generated by intramembranous ossification with subsequent endochondral ossification forming the condyle. The identities and properties for SSCs in these two sites remain unknown. Here, we use genetic lineage tracing in mouse to identify cells expressing the Hedgehog responsive gene *Gli1,* which is thought to mark the tissue resident SSCs. We track the *Gli1+* cells, comparing cells within the perichondrium to those in the periosteum covering the mandibular body. In juvenile mice, these have distinct differentiation and proliferative potential. We also assess the presence of *Sox10+* cells, thought to mark neural crest stem cells, but find no substantial population associated with the mandibular skeleton, suggesting that *Sox10+* cells have limited contribution to maintaining postnatal mandibular bone. All together, our study indicates that the *Gli1+* cells display distinct and limited differentiation capacity dependent on their regional associations.

## Graphical abstract

Gli1‐positive skeletal stem cells have reported in association with calvarial bones. In the mandible, most of the bone is formed via intramembranous ossification, while the condyle is uniquely formed via endochondral ossification. Here, explore the distribution of Gli1‐positive cells in the mandible and propose that there are both osteogenic and chondrogenic sub‐populations associated with maintenance of postnatal mandibular bone. We further find that no association of Sox10 positive cells with these tissues.

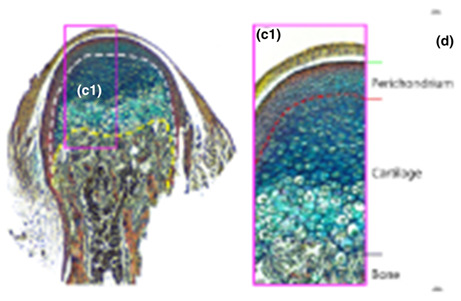

## 1 INTRODUCTION

The human skeletal system has two developmental origins: mesoderm, which gives rise to non‐craniofacial bones such as the vertebral column and appendicular skeleton, and neural crest, which contributes to most of the craniofacial bones (Olsen et al., 2000). There are also two distinct ossification programs. Most of the non‐craniofacial bones are formed by endochondral ossification while most of the craniofacial bones are formed by intramembranous ossification. During endochondral ossification, a cartilaginous template is first formed, followed by gradual replacement of the cartilage with bone. During intramembranous ossification bony tissue is directly formed, with no cartilage is observed (reviewed in (Berendsen & Olsen, 2015)). Among all the craniofacial bones, the mandible is special in that it is formed by both modes of ossification. The mandibular body (alveolar bone and ramus) is formed through intramembranous ossification, while the condyle is formed by endochondral ossification (Parada & Chai, 2015).

Skeletal stem cells (SSCs) are a multi‐potent cell population that gives rise to bone and cartilage. Later in life, tissue‐resident skeletal stem cells are key players in bone homeostasis. Advances in recent years have provided some insights into sub‐populations of SSCs within different anatomical domains. In the long bone, there are at least two distinct niches for SSCs, one within the growth plate and one within the periosteum. When transplanted into the renal capsule, the growth plate‐resident SSCs follow a typical endochondral ossification pattern, with generation of bone, cartilage, and stromal tissue supporting hematopoiesis (Chan et al., 2009, 2015). In contrast, the periosteal SSCs follow an intramembranous ossification pattern: only bone is generated without cartilage or haematopoietic elements (Debnath et al., 2018). Similarly, in the craniofacial region, cultures derived from the condylar perichondrium generated both cartilage and osseous matrix (Silbermann et al., 1987). However, cells isolated from the calvarial periosteum or suture only generated bone tissue in transplantation assays (Debnath et al., 2018; Maruyama et al., 2016). In situ analysis showed that the mesenchymal cells in the periosteum of the mandibular body only express *Runx2*, indicating osteogenic capacity, while mesenchymal cells in the condyle co‐express *Runx2* and *Sox9*, suggesting both osteogenic and chondrogenic differentiation (Shibata et al., 2006; Yamashiro et al., 2004).

These data suggest that SSCs associated with the mandible may have distinct differentiation properties dependent on their association with the perichondrium versus the periosteum. However, to date, SSCs in the craniofacial region have been poorly studied due to the lack of a good stem cell marker. Recently, several mouse *Cre‐*driver lines have been developed, including *Gli1* (Zhao et al., 2015), *Axin2* (Maruyama et al., 2016) and *Prrx1* (Wilk et al., 2017) lines, which are suggested to label craniofacial stem cells. Here, we used the *Gli1‐Cre* ^*ERT2*^ mouse line to investigate whether *Gli1* marks the SSCs population in the mandibular condyle and periosteum. We found that *Gli1+* cells distribute within the periosteum and some specific areas in the condyle. Further, we assessed their differentiation ability and found that it is regionally restricted. The condylar *Gli1+* cells possess both osteogenic and chondrogenic potential while the periosteal‐associated cells only have osteogenic potential.

Because the mandible is of neural crest (NC) origin, we also considered the possibility that *Gli1*+ cells expressed *Sox10*, a master regulator of the neural crest lineage. *Sox10*+ NC cells (NCCs) are found not only in the embryonic and early postnatal stages but have also been hypothesized to be tissue‐resident adult stem cells. They have been found in various NC‐derived organs such as: cornea (Yoshida et al., 2006), hard palate (Widera et al., 2009), oral mucosa (Marynka‐Kalmani et al., 2010), dental pulp (Abe et al., 2012) and periodontal ligament (Kaku et al., 2012; Techawattanawisal et al., 2007). Furthermore, the NCCs generally showed broader differentiation capacity compared with SSCs, raising the possibility that *Gli1*+ SSCs might be a subset of *Sox10*+ stem cells. To test this, we utilized a *Sox10::Cre* ^*ERT2*^ mouse line and found no clear evidence for the involvement of *Sox10* positive cells in mandibular development. In conclusion, while *Gli1*+ cells are able to contribute to both intramembranous and endochondral ossification, these cells display distinct and limited differentiation capacity dependent on their regional associations.

## 2 MATERIALS AND METHODS

### 2.1 Mouse lines and methods

Wild‐type mice were outbred CD1 strain. *Gli1::creERT2/+* (MGI:3053957) (Ahn & Joyner, 2004) or *Sox10‐icre/+* mice (MGI:5301107) (Laranjeira et al., 2011) were crossed with mice carrying a cre‐responsive reporter *Rosa* ^*mTmG/mtmG*^ (MGI:3716464) (Muzumdar et al., 2007) to generate *Gli1::creERT2/+;Rosa* ^*mTmG*^ and *Sox10‐icre/+;Rosa* ^*mTmG/+*^ mice. Mice were treated by intraperitoneal injection tamoxifen at a dosage of 1.5 mM/g of body weight at time points indicated. The tamoxifen (Sigma T5648) was suspended in 10% ethanol and 90% corn oil (Sigma C8267) to reach a concentration of 20 mg/mL. All animal experiments conform to ARRIVE guidelines. Experiments were ethically approved and performed in accordance with UK Home Office License P8D5E2773 (KJL).

### 2.2 Histology

Samples were dissected and fixed in 4% paraformaldehyde overnight at room temperature, and then decalcified in 10% Formic acid for 48 h. Decalcified samples were dehydrated in 25%, 50%, 75%, 100% ethanol stepwise at room temperature. Samples were then embedded according to standard protocols and sectioned at 8 μm thickness using a microtome (Leica CM1850). Haematoxylin and eosin (H&E) staining and PicoSirius Red trichrome staining were performed as previously described (Tabler et al., 2013). Images were taken using a Nikon Eclipse Ci.

### 2.3 Immunofluorescent staining

For immunofluorescent staining on slides. The 8 μm sections were dewaxed, rehydrated to PBS. Antigen‐retrieval was performed in heated citrate buffer. Following antigen‐blocking, the sections were incubated with primary antibody at 4°C overnight. Primary antibodies used for GFP (ab13970, Abcam), Sox10 (SC‐365692, Santa Cruz), Runx2 (ab192256, Abcam), Sox9 (ab185230, Abcam), OPN (ab69498, Abcam), ColX (ORB10444, Biorbyt). All the primary antibodies were diluted in 1:500. Then after washing for 3 times the next day, the sections were incubated with 1:500 secondary antibodies (Goat anti‐chicken 488 (Life Technologies A11039)), Goat anti‐mouse 488 (Life Technologies A11001), or Goat anti‐rabbit 568 (Life Technologies A11011) for 90 min at room temperature, followed by three washes. At last, sections were mounted with DAPI mounting medium (ab104139, Abcam).

For immunofluorescent staining on cells, the cells were fixed with 4% paraformaldehyde at 4°C overnight. Then the cells were washed with 0.05% PBST 3 times. After blocking, the cells were incubated with primary antibody at 4°C overnight. Primary antibody used: anti‐GFP (ab13970, Abcam, 1:500) followed by a goat anti‐chicken 488 (Life Technologies A11039) After washing for 3 times the next day, the cells were stained with DAPI.

Confocal images were acquired using Leica SP5 confocal. Images were processed with Image J software. Representative images from three independent samples or experiments are shown in the figures.

### 2.4 Explant culture

The mandibular condyle and periosteum were obtained from P14 *Gli1‐cre* ^*ERT*^ *;Rosa* ^*mTmG*^ mice, and cultured in 24‐well plate. Media consisted of alpha‐MEM (Lonza, BE02‐002F), supplemented with 10%, FBS, osteogenic batch tested (Gibco, 10,270, Lot 41F8146K), 1% ABAM (Sigma, A5955), and 1% Glutamine (Sigma, G7513). 2 μg/mL 4‐OH‐Tamoxifen (Sigma, H7904) was added to the media the first day and removed after 48 h. The medium was changed every other day.

### 2.5 Statistical analysis

Every experiment was repeated at least 3 times independently. For cell counting, we selected 3 slices from each individual animal with a total of 9 slices. Data were presented as mean ± SEM deviation, p‐values were determined by unpaired *t*‐test and graphs made using Graphpad (Prism Software).

## 3 RESULTS

### 3.1 Gli1+ cells reside within both the perichondrium and the periosteum but show regional expansion ability

**FIGURE 1:**
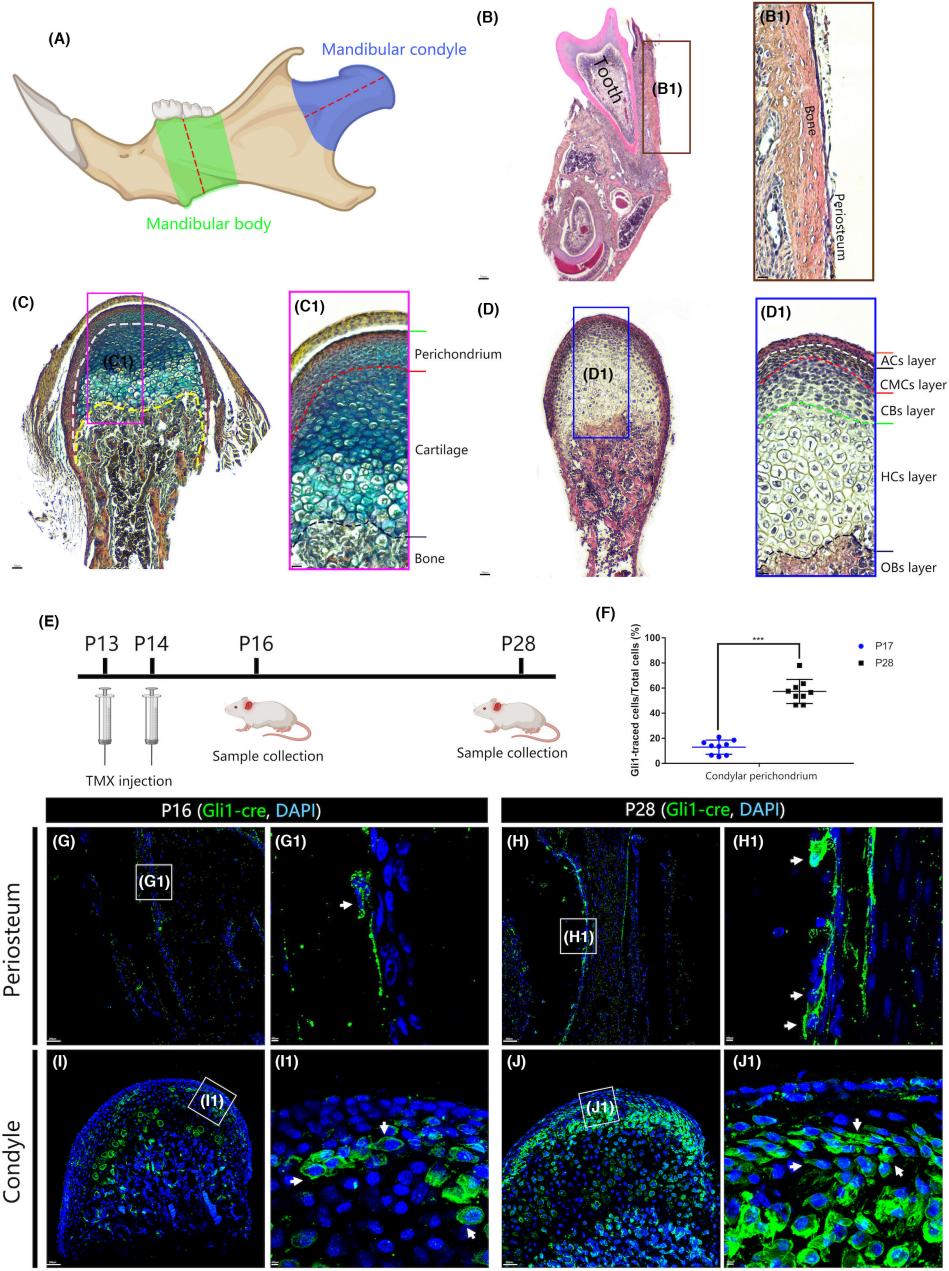
*Gli1+* cells reside within the perichondrium and the periosteum but show different expansion ability. Histological features of the periosteum (mandibular body) and the perichondrium (A–D), Gli1‐cre lineage tracing experiments (E–J). Mandibles from P16 wild‐type mice were collected, sectioned and stained with H&E staining and (B, D) PicoSirius Red trichrome staining (C). B_1_, C_1_ and D_1_ are high‐magnification images of the boxes in B, C and D respectively. Two parts of the mandibles are sectioned, the condyle (A, blue region) and the periosteum (A, green region); sections are indicated (A, red dashed lines). The periosteum covering the mandibular body consists of two to three cell layers. (B, B_1_). In the condyle, three tissue structures can be identified: the perichondrium, the cartilage and the bone (C, C_1_). From proximal to distal directions, six cellular layers can be identified in the H&E staining (D, D_1_). The articular cells (ACs) layer, condensed mesenchymal cells (CMCs) layer, hypertrophic chondrocytes (HCs) layer, chondroblasts (CBs) layer and osteoblasts (OBs) layer (D, D_1_). The perichondrium consists of ACs and CMCs layers; the cartilage consists of CBs and HCs layers; the bone consists of OBs layer. *Gli1‐cre/+; Rosa* ^*mTmG*^ mice were treated with tamoxifen at postnatal day 13 (P13) and P14, mandibles were collected at P16 and P28 (E). Anti‐GFP (green) and nuclei (DAPI, blue). G_1_, H_1_, I_1_, and J_1_ are high‐magnification images of the boxes in G, H, I and J, respectively. In the periosteum, few Gli1+ cells displayed a scattered distribution at P16 (G, G_1_ white arrow). After 14 days tracing, the number of *Gli1*‐traced cells are scarce with some cells are clustered (H, H_1_ white arrows). In theperichondrium, *Gli1+* cells only accounted for 12.9% of the total number, and this ratio increased to 57.3% after 14 days' tracing (F). Representative images were shown in (I, J). Scale bars (B–D): 10 μm; (G–J): 200 μm; (G_1_–J_1_): 400 μm. White arrows: Gli1‐cre traced cells. Data are shown as mean ± SEM, ***p<0.001.

To demonstrate the histological structures of the mandible, we collected the mandible and focused on the mandibular periosteum and the condyle from postnatal day 16 (P16) wild‐type mice (schematic shown in Figure 1A). We focused specifically on the periosteum covering the surface of the mandibular body (Figure 1A, green region). The tissue structure of the periosteum is quite simple, consisting of two or three cell layers (Figure 1B, B_1_). The tissue structure in the condylar region (Figure 1A blue region) is more complex than the periosteum. We performed trichrome staining, which includes Alcian Blue (labeling glycosaminoglycans in cartilage) and PicoSirius Red (labeling calcified bone) (Figure 1C, C_1_). Our staining showed three distinct anatomical domains: the periochondrium, cartilage and bone (marked in Figure 1C1). Further, using haematoxylin and eosin (H&E) staining (Figure 1D, D_1_) we identified 6 cellular layers based on their morphology (Mizoguchi et al., 2013). The perichondrium consists of articular cell (ACs) and condensed mesenchymal cells (CMCs) layers; the cartilage consists of chondroblast (CBs) and hypertrophic chondrocytes (HCs) layers; the bone consists of osteoblasts (OBs) layer. (Figure 1D, D_1_). These structures are similar to a typical endochondral growth plate, with the CMC, CB and HC layers correspond to resting, proliferative and hypertrophic zones.

We used a lineage labeling approach to determine the distribution of *Gli1+* cells within the mandible. To do this, we generated mice carrying Cre‐recombinase driven by a *Gli1* promoter combined with Cre‐responsive reporter *Rosa* ^*mtmg*^ (*Gli1::creERT2/+;Rosa* ^*mTmG*^). In these mice, all cells express membrane red fluorescent protein (mRFP) until the administration of tamoxifen (TMX) activates Cre in cells expressing the *Gli1* promoter, which then express membrane green fluorescent protein (mGFP). This approach provides a timed snapshot of Gli1‐positive cells as well as the ability to follow the progeny of these cells.

As shown in Figure 1E, tamoxifen (TMX) was administrated at P13 and P14. Subsequently, samples were collected at P16 or P28 as indicated. We found that in the periosteum, only a few *Gli1*‐traced cells displayed a scattered distribution at P16 (G, G_1_ white arrows). After 14 days of tracing, the number of *Gli1*‐traced cells was still scarce with some cells clustered (H, H_1_ white arrows).

In the perichondrial region the *Gli1*‐traced cells are mainly located at the CMCs layer (Figure 1I, I_1_). After 14 days of tracing, the condylar *Gli1+* cells expanded quickly and gave rise to the majority of the perichondrial cell populations (Figure 1J, J_1_). To verify these results, we then counted the percentage of the *Gli1*‐traced cells/total cells. Our data showed that the proportion at the beginning of tracing (P16) was 12.9% 14 days later, the proportions increased to 57.3% (Figure 1(F)) These data suggest that *Gli1+* cells in the condylar region are highly proliferative while those in the periosteum are less proliferative.

### 3.2 No obvious difference in the behavior between the perichondrium‐ or periosteum‐origin Gli1+ cells

**FIGURE 2:**
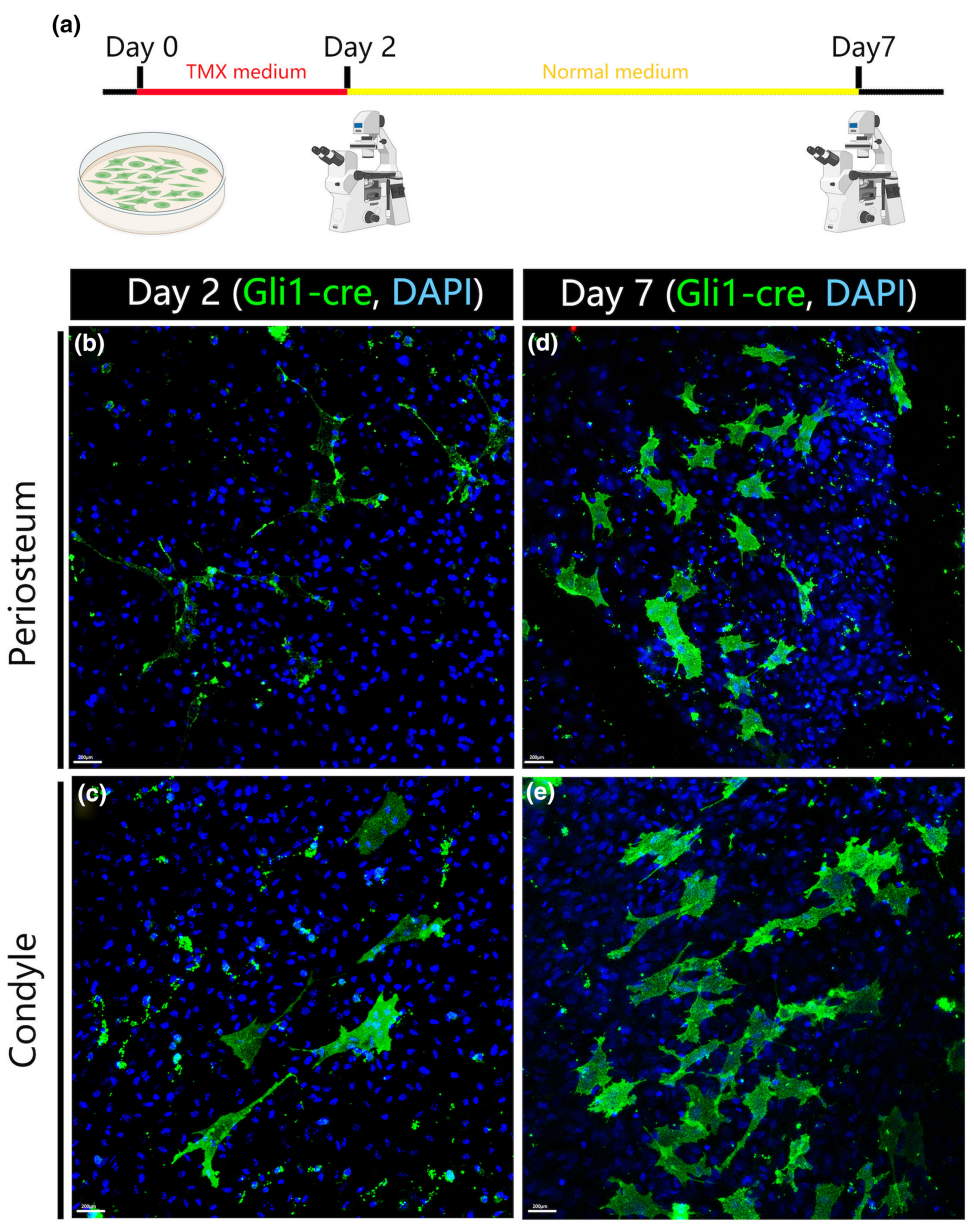
Periosteal and perichondrial Gli1+ cells showed similar behavior in vitro. Periosteal and condylar cells from P16 *Gli1‐cre/+; Rosa* ^*mTmG*^ mice were dissected and cultured for 7 days. Tamoxifen was added to the media from day 0 and removed at day 2 (a). Cells emerging from the explants were fixed and stained for anti‐GFP (green) and nuclei (DAPI, blue). The morphology of the both periosteal and perichondrial Gli1‐traced cells is polymorphic but not typical fibroblasts‐like (b, c). Scale bars: 200 μm.

To investigate the in vitro behavior of *Gli1+* cells, we explanted cultured condylar and periosteal cells from P16 *Gli1::creERT2/+;Rosa* ^*mTmG*^ mice. Induction medium was administrated at day 0; the induction medium was then removed and changed into normal medium from day 2. We found that there is no obvious difference in the behavior between the condyle‐origin or periosteum‐origin Gli1+ cells. The morphology of the cells is polymorphic but not typically fibroblast‐like (Figure 2).

### 3.3 Gli1+ cells in different regions have different differentiation potential

As noted in the introduction, the condyle and mandibular body follow different ossification programmes. The mandibular body is formed by intramembranous ossification early in embryogenesis, followed later by formation of the condyle via endochondral ossification. This raises the possibility that osteogenesis in the condylar region depends on a chondrocytic or osteochondrocytic precursor, while in the main mandible body, osteogenesis and chondrogenesis may be uncoupled. While *Gli1+* cells are evident in both regions, it is unclear whether these regional cells have equivalent differentiation capacity.

**FIGURE 3:**
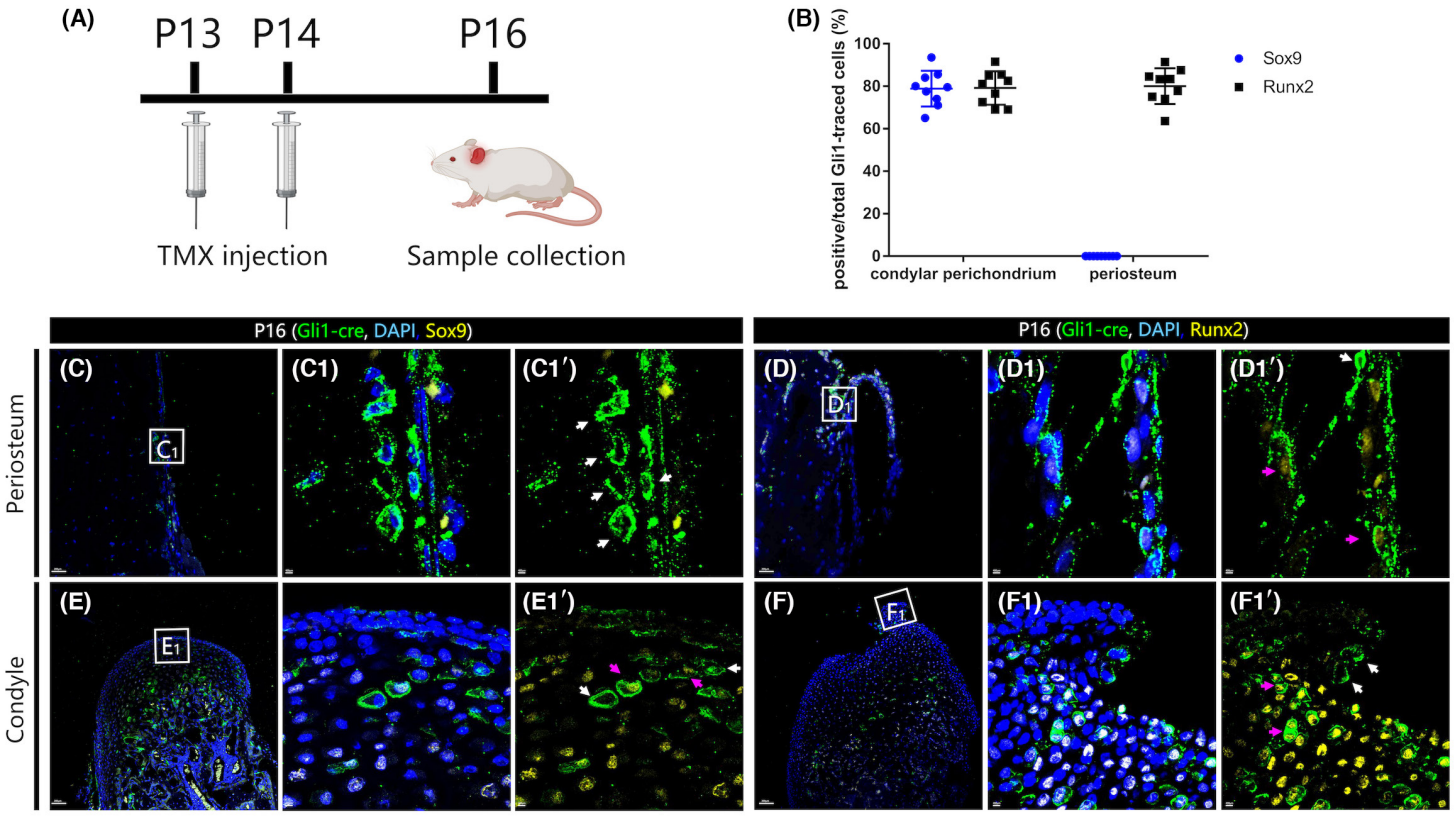
*Gli1+* cells in the perichondrium and periosteum have distinct differentiation potential. *Gli1‐cre/+; Rosa* ^*mTmG*^ mice received tamoxifen at P13 and P14, and collected at P16 (A). Antibody staining for anti‐GFP (green), nuclei (DAPI, blue) and anti‐Runx2 or Sox9 (yellow). C_1_, D_1_, E_1_ and F_1_ are high‐magnification images of the boxes in C, D, E and F respectively. C_1_’, D_1_’, E_1_’ and F_1_’ were processed without DAPI channel. In the periosteum, no *Gli1+* cell was positive for Sox9 (C1’ white arrows). *Runx2‐*positive (D_1_’ pink arrowheads) and *Runx2‐*negative (D_1_’ white arrowheads) *Gli1+* cells are seen in the periosteum (D, D_1_, D_1_’). Of the *Runx2* positive population comprise 80.1% of the total periosteal *Gli1+* cells (B). In the perichondrium, we found that *Sox9* positive (E_1_’ pink arrows) and negative (E_1_’ white arrows) *Gli1 +* cells coexisted. A similar result was found in Runx2 stainings, Runx2 positive (F1’ pink arrows) and negatice (F1’ white arrows) also coexisted. Of the *Gli1+* cells in periochondrium 78.9% are positive for Sox9, 79.2% are positive for Runx2 (B). Scale bars: 200 μm. Data are shown as mean ± SEM.

To examine this, we collected samples from P16 *Gli1::creERT2/+;Rosa* ^*mTmG*^ mice, 2 days after TMX induction (Figure 3A). We immuno‐stained with anti‐GFP (recognizing *Gli1+* cells), Runx2 (which triggers osteogenic differentiation) and Sox9 (a regulator of chondrogenic differentiation). We found that in the periosteum, no *Gli1*‐traced cells were found to be Sox9 positive (Figure 3C), but the majority of these cells are Runx2 positive (Figure 3D). In contrast, in the perichondrium, most *Gli1*‐traced cells are positive for both Sox9 and Runx2 protein (Figure 3E,F) though there were a few negative cells (Fig. 3E_1_’, F_1_’ white arrows). To verify these results, we then counted the percentage of Sox9 or Runx2 positive *Gli1*‐traced cells compared to the total *Gli1*‐traced cells in each region. We found that in the periosteum 80.1% of the *Gli1*‐traced cells are positive for Runx2 but none were positive for Sox9 (Figure 3B). In the perichondrium, 78.9% are positive for Sox9, 79.2% are positive for Runx2 (Figure 3B). Due to technical limitations of our genetic cross, we were unable to perform immunofluorescence imaging for Sox9 and Runx2 in the same sections. Nevertheless, this suggests that *Gli1+* cells associated with the perichondrium have both osteo‐ and chondrogenic capacity.

### 3.4 Perichondrial Gli1+ cells give rise to both chondroblasts and osteoblasts

**FIGURE 4:**
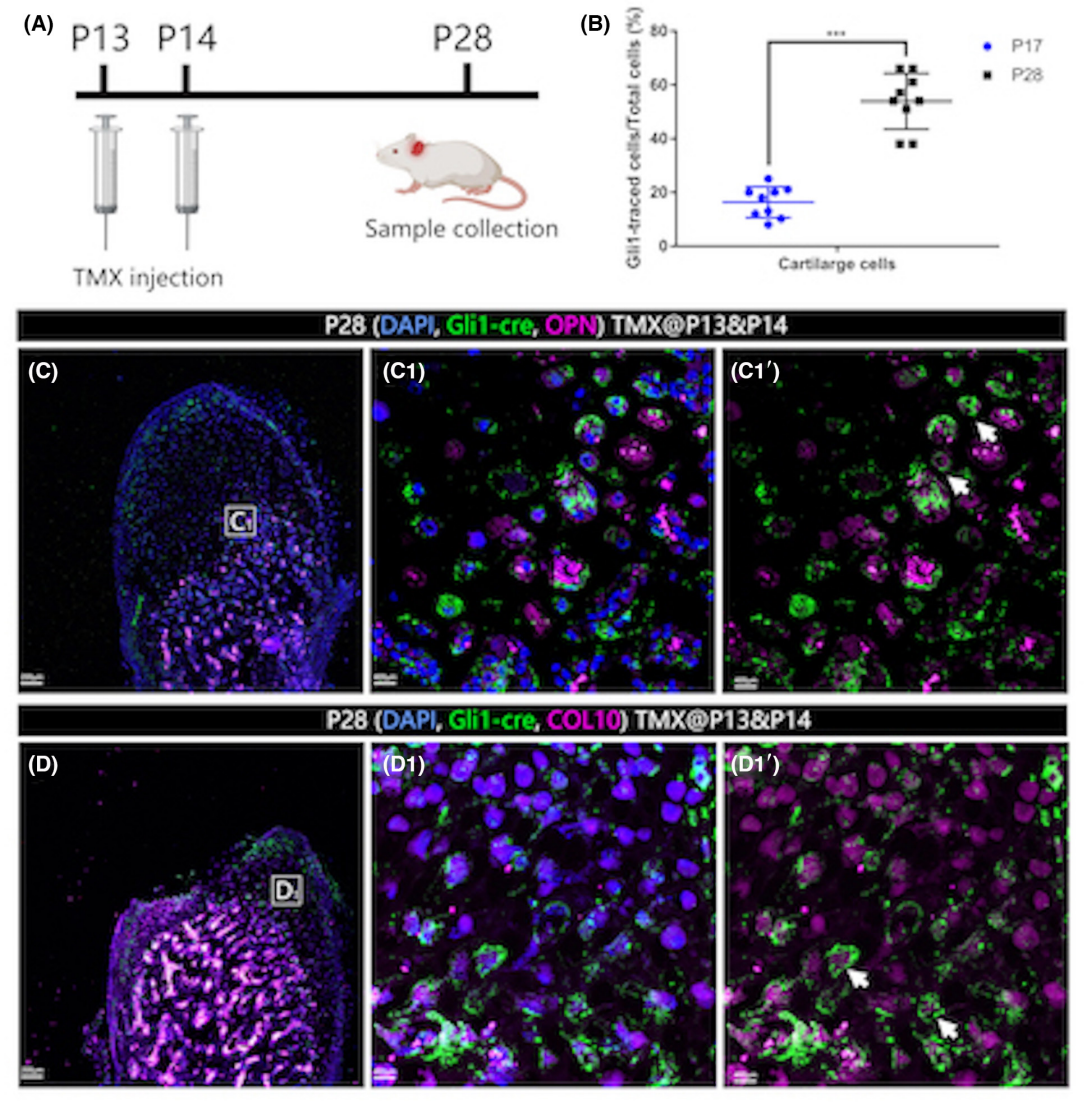
Condylar *Gli1+* cells give rise to chondrocytes and osteoblasts. *Gli1‐cre/+;Rosa* ^*mTmG*^ mice received tamoxifen atpostnatal day 13 (P13) and P14, mandibles were collected at P28 (A). Condyles were fixed and stained for Anti‐GFP (green) nuclei (DAPI, blue) and Anti‐OPN/ColX (magenta). Osteopontin (OPN) is a mature osteoblast marker and CollagenX (ColX) is a mature chondrocyte as well as osteocyte marker. C_1_ and D_1_ are high‐magnification images of the boxes in B and C respectively; C_1_’ and D_1_’ were processed without DAPI channel We found that in 14 days' tracing Gli1‐cre positive cell population ratio increased from 16.3% to 53.9% (B) in cartilage zone meanwhile some OPN+ osteoblasts (C) beneath the cartilage were traced (C_1_’ white arrows). Scale bars (C, D): 200 μm; (C_1_, C_1_’, D_1_, D_1_’): 400 μm.

In order to test whether the *Gli1+* cells could eventually give rise to both chondroblasts (CBs) and osteoblasts (OBs), we collected samples from P28 *Gli1::creERT2/+;Rosa* ^*mTmG*^ mice, 14 days after TMX induction (Figure 4A). We used antibody staining to identify GFP (for the *Gli1+* lineage), osteopontin (OPN, a mature osteocyte marker) and collagen X (ColX, a mature chondrocyte/osteocyte marker). We found that at 14 days tracing the ratio of Gli1‐cre positive cell populations increased from 16.3% to 53.9% (Figure 4B,D) in condylar cartilage zone meanwhile the proportion of Gli1‐cre positive OPN+ osteocytes increased from 9.3% to 39.1% (Figure 4C).

We also examined the periosteal *Gli1*‐traced cells. We did not see any labeled osteocytes under the periosteum, likely reflecting the slow turnover of cortical bone in the mandible.

### 3.5 Sox10+ cells do not contribute to postnatal periosteal and condylar development

**FIGURE 5:**
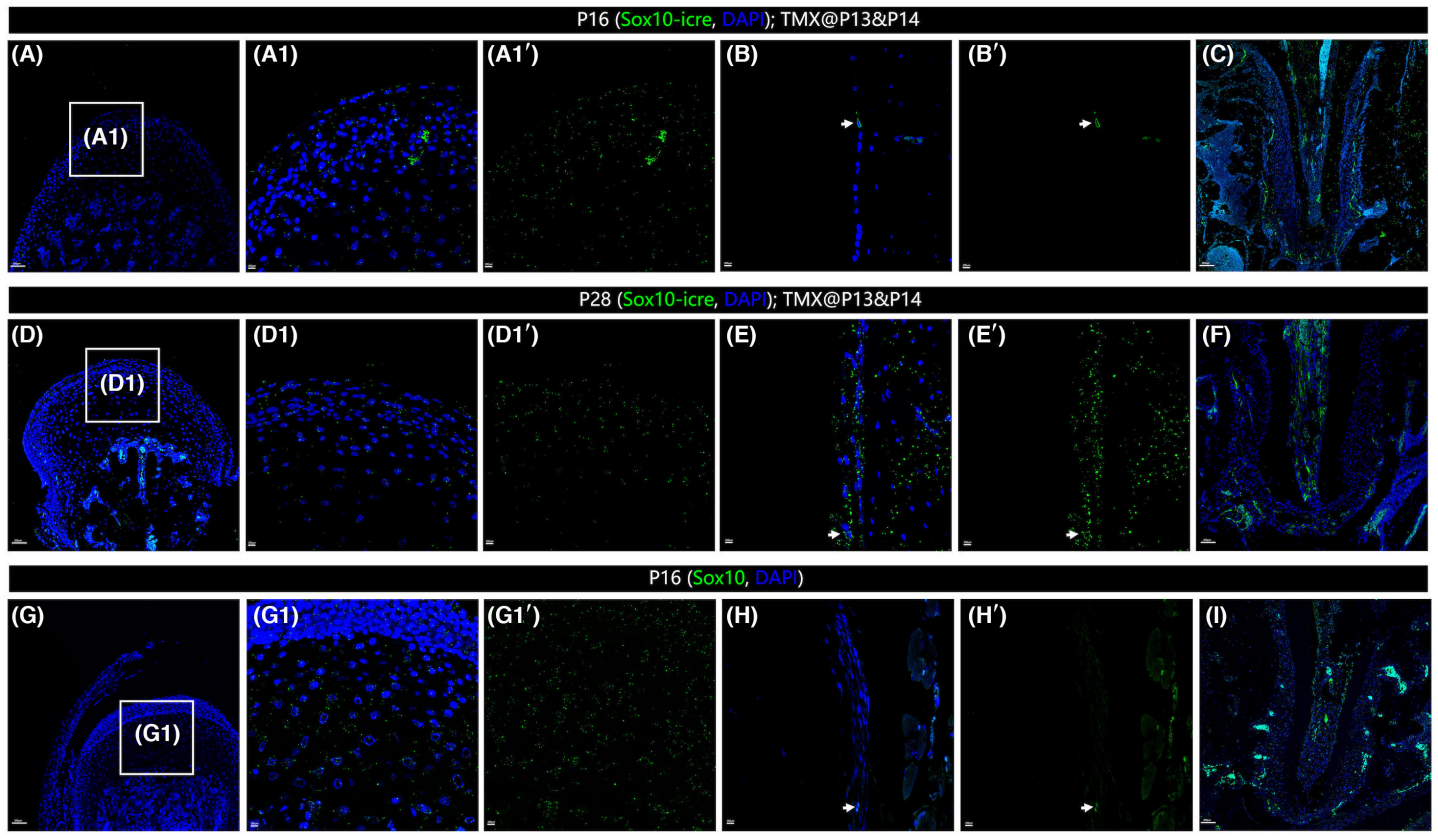
*Sox10+* cells are not associated with postnatal periosteum and condyle. *Sox10‐icre/+; Rosa* ^*mTmG*^ mice received tamoxifen at postnatal day 13 (P13) and P14, mandibles were collected at P16 and P28, samples were stained for anti‐GFP (green) and nuclei (DAPI, blue). For Sox10 immunostaining samples were collected at P16 from wild‐type CD1 animals, then stained for Sox10 (green) and nuclei (DAPI, blue). A, D and G are representative images for the condyle; B, E and H for the periosteum; C, F and I for the teeth. A_1_, D_1_, G_1_ and H_1_ are high‐magnification images of the boxes in A, D, G and H respectively. A_1_’, B_1_’, D_1_’, E_1_’, G_1_’ and H_1_’ were processed with removal of nuclei. In the condyle region, no cell was traced either at P16 (A, A_1_, A_1_’) or P28 (D, D_1_, D_1_’). This is in coincidence with Sox10 antibody staining: no cell is positive for Sox10 (G, G_1_, G_1_’). In the periosteum, very rarely can we see a single Sox10‐traced cell at P16 (B, B′ white arrowhead). After 14 days' tracing, in most slices no GFP cell was observed. Although a single GFP cell could be seen (E, E' white arrowhead), no clones were found. This is in coincidence with Sox10 antibody staining, where we saw few Sox10 positive cells (H, H′) on the periosteum. As a positive control, we show Sox10 in teeth (C, F, I). Scale bars: 200 μm.

As noted above, the mandibular skeleton is derived from the neural crest lineage. In the embryo, expression of *Sox10* is associated with neural crest multipotency (Kim et al., 2003) Later in life, significantly higher expression levels of *Sox10* have been found in the mandibular periosteum when compared to femur‐associated periosteum (Ichikawa et al., 2015). We designed two strategies to investigate the possibility that postnatal *Sox10+* cells may also contribute to mandibular development and maintenance. First, we performed a lineage tracing experiment to parallel the *Gli1*‐tracing experiments described above. For this experiment, the *Sox10‐icre/+;Rosa* ^*mTmG/+*^ mice were treated with tamoxifen at P13 and P14 and mandibles were collected at P16 and P28. Samples were stained for anti‐GFP (green, labeling the *Sox10*‐traced cells) and nuclei (DAPI, blue). In parallel, samples were collected at P16 from wild‐type animals; these were then immune‐stained for anti‐Sox10 (green) and nuclei (DAPI, blue). To our surprise, we did not find any *Sox10*‐expressing cells in the condylar region either by lineage tracing (Figure 5A,D) or by Sox10 antibody staining (Figure 5G). In the periosteal region, we very rarely saw a few *Sox10*‐traced cells (Figure 5B', E' white arrowheads) or Sox10 positive cells (Figure 5H' white arrowheads). As a positive control, we found *Sox10*‐positive cells in the teeth (Figure 5C,F,I), which had been previously described. Our results showed that no condylar cell was Sox10 positive by P14 including the *Gli1+* cells; very few cells in the periosteum are positive for Sox10. Because the number of *Sox10+* cells was exceedingly small and after 14 days tracing the Sox10‐traced cells did not proliferate to form clones, we speculate that the contribution of *Sox10+* cells to mandibular development at least in a bone/cartilage generation manner from P14 is limited.

## 4 DISCUSSION

In this study, we exploited the unique properties of the mandible to test our hypothesis that there are distinct subtypes of *Gli1+* cells associated with endochondral and intramembranous bone. First, we identified *Gli1+* cells as a population of osteochondral progenitors contributing to the mandible, especially during condylar development. Second, we found that *Gli1+* cells may have distinct differentiation properties, which are associated with the perichondrium versus the periosteum. Third, we did not find clear evidence for the involvement of *Sox10+* cells in mandibular development.

The Hedgehog (Hh) signaling pathway is a highly conserved pathway playing multiple roles during bone development (Yang et al., 2015). As noted above, Hh responsive cells (expressing *Gli1*) have been proposed to mark SSCs in both long bones and cranial bones. Gli1+ cells isolated from bone marrow have the ability to differentiate to bone, cartilage and adipocytes (Shi et al., 2017). In the long bone, Gli1+ cells were found to reside in SSCs niches like the growth plate, periosteum and bone marrow, they gave rise to osteogenic lineages along the longitudinal axis (Haraguchi et al., 2018). However, a population beneath the growth plate was found to express *Runx2* but not *Sox9* (Shi et al., 2017), suggesting that these cells are limited to the bone lineage and lack chondrogenic capacity. In the cranial region, the adipogenic differentiation ability of the sutural Gli1+ cells was much weaker than that of the bone marrow Gli1+ cells (Zhao et al., 2015). These studies showed that not all Gli1+ cells are equivalent.

The periosteum and perichondrium are both critical for mandibular development mediating intramembranous and endochondral ossification respectively. This led us to consider whether SSCs in these positions are naturally different. Here, we found that Gli1+ cells reside in both sites (Figure 1). Our finding of Gli1+ traced cells in the CMC layer is consistent with a role for this layer as the growth center for condylar development (Hinton et al., 2015). The *Gli1+* cell population in the CMC layer is highly proliferative. By 14 days of tracing, the population of perichondrial Gli1+ cell population increased by more than 4 times. Meanwhile they generated more than half of the cartilage cells beneath the perichondrium. We can predict that given longer tracing times the perichondrial Gli1+ cells will fill the condyle. Previous in situ studies only demonstrated that the perichondrium expressed *Runx2* and *Sox9* mRNA but did not assess at the individual cell level (Shibata et al., 2006). Because our study shows that most Gli1+ cells were positive for Runx2 (79.2%) or Sox9 (78.9%) (Figure 3), it is likely that some proportion of Gli1+ cells express both Runx2 and Sox9 at the same time, indicating osteochondrogenic capacity. This still needs to be formally validated.

We also found that the majority of Gli1+ cells in the HC layer are positive for Runx2. These cells could be resident SSC‐like cells or could be responding to local Hedgehog signaling. Indian Hedgehog (Ihh) positive cells are also found at the cartilage‐bone interface (Long et al., 2019). Beyond its basic function in stimulating the expression of parathyroid hormone‐related peptide (PTHrP) (Kindblom et al., 2002; Vortkamp et al., 1996), Ihh is reported to promote chondrocyte hypertrophy and matrix mineralization directly. Ihh can induce *Runx2* expression in responsive chondrocytes (Amano et al., 2014) This could explain why the Gli1+ in HCs layer are positive for the osteogenic factor Runx2.

Another possibility is that these cells are in a transient SSC‐like state. Hypertrophic chondrocytes were suggested to turn over due to apoptosis. However, some long‐bone chondrocyte‐tracing experiments have suggested that hypertrophic chondrocytes that do not undergo apoptosis can become BMSCs or osteoblasts (Ono et al., 2014; Park et al., 2015; Yang et al., 2014), and Jing et al. stated that about 80% of the osteoblasts in the condylar neck region were of chondrocyte origin (Jing et al., 2015). So, these *Gli1*+ cells may be differentiated chondrocytes that then undergo dedifferentiation to become transient SSCs. Nevertheless, we observed that condylar Gli1+ cells are proliferative, most of them are positive for both Runx2 and Sox9 thus supporting the idea that the Gli1+ cells in the condylar region are dual‐potential SSCs.

In contrast, when we examine the periosteum, the *Gli1+* cell population is scarce. After 14 days tracing, clustered cells could be found. These cells did not expand as robustly as the condylar ones. The reason may lie in the growth rate of mandibular body, which is lower than the condyle in puberty (Gomes & Lima, 2006). Generally, the remodelling rate of cortical bone is also lower than that of cartilage. Furthermore, in all the samples checked we did not find any *Gli1+* cells positive for Sox9, while most were positive for Runx2. This supports previous studies, which were performed by assessing mRNA expression (Yamashiro et al., 2004), and is consistent with the idea that the Gli1+ cells in the periosteum are uni‐potential SSCs.

Finally, our last goal is to investigate whether mandibular Gli1+ cells have an associated Sox10 signature, reflecting their neural crest origins (Figure 5). We have previously demonstrated that craniofacial osteoblasts isolated from neural crest‐tissues, such as frontal bone and dura mater, have higher intrinsic osteogenic capacity than those isolated from mesoderm‐derived parietal bone (Doro et al., 2019). This raised the possibility that neural crest cells might contribute specifically to homeostasis of the manible bone. However, we found that very few Sox10+ cells reside in association the periosteum or the condyle, despite robust persistence of Sox10 protein found in tooth cells. This may reflect the transience of cells in mouse teeth, which undergo continuous turnover.

In summary, we suggest that local Gli1+ cells show SSCs activities. These *Gli1+* cells are able to contribute to both intramembranous and endochondral ossification and display distinct and limited differentiation capacity dependent on their regional associations.

## Data Availability

The data that support the findings of this study are available from the corresponding author upon reasonable request.
